# Hepatitis B surface antigen positivity is associated with poor short- and long-term outcomes in patients with diffuse large B-cell lymphoma who received CHOP or R-CHOP

**DOI:** 10.3389/fimmu.2024.1324113

**Published:** 2024-01-22

**Authors:** Zhumei Zhan, Wei Guo, Xin Wan, Bowen Wang, Jia Li, Haotian Wang, Zhe Li, Yuhua Huang, Ken H. Young, Ou Bai

**Affiliations:** ^1^Department of Hematology, The First Hospital of Jilin University, Changchun, China; ^2^Department of Pathology, Sun Yat-sen University Cancer Center, Guangzhou, China; ^3^Division of Hematopathology, Department of Pathology, Duke University Medical Center and Duke Cancer Institute, Durham, NC, United States

**Keywords:** HBV infection, HBsAg, diffuse large B-cell lymphoma, progression, next-generation sequencing

## Abstract

**Objective:**

The development of diffuse large B-cell lymphoma (DLBCL) is closely related to the host infection status. China is a highly endemic area for hepatitis B virus (HBV) infection. It is not clear whether HBV infection has a consistent effect on the prognostic implications of patients with DLBCL in different treatment settings.

**Materials and methods:**

We conducted a cohort study of 692 patients with DLBCL receiving three or more cycles of treatment with a CHOP or R-CHOP regimen from the First Hospital of Jilin University between July 2011 and July 2022. The patients were divided into two groups based on their hepatitis B surface antigen (HBsAg) status: HBsAg-positive (n = 84, 12.1%) and HBsAg-negative (n = 608, 87.9%) groups. Tumor specimens from 180 patients with primary DLBCL were collected for next-generation sequencing (NGS).

**Results:**

The HBsAg-positive group had more frequent abnormal liver function (P = 0.003), hypoalbuminemia (P < 0.001), incidence of > 2 extranodal organs (P = 0.011), and spleen involvement (P < 0.001) than the HBsAg-negative group. HBsAg-positive patients had lower complete response (CR) and overall response rates (ORR) rates (all the p values < 0.05), in either the CHOP group or R-CHOP group. Among patients receiving R-CHOP, the rates of disease progression within 12 and 24 months were higher in the HBsAg-positive group than in the HBsAg-negative group (P=0.018, P=0.029). However, no significant difference in disease progression was observed between HBsAg-positive and HBsAg-negative patients in the CHOP group(P > 0.05). HBsAg positivity (OS: HR [95% CI] = 2.511 [1.214-5.192], P = 0.013) was only associated with poorer OS in the CHOP group. Whereas in the R-CHOP group, HBsAg positivity was associated with both poorer OS and PFS (OS: HR [95% CI] = 1.672 [1.050-2.665], P = 0.030; PFS: HR [95% CI] = 1.536 [1.013-2.331], P = 0.043). Additionally, HBsAg-positive patients with DLBCL also had a higher prevalence of mutations in MYC, ATM, PTPN6, and epigenetically regulated genes.

**Conclusion:**

These findings suggest that HBsAg-positive DLBCL patients may represent a distinct subgroup with a poorer prognosis. The standard therapies may be insufficient and new therapeutic strategies should be developed based on a better understanding of the underlying mechanisms of chemoresistance.

## Introduction

Hepatitis B virus (HBV) infection is a worldwide health problem. The World Health Organization (WHO) estimates that two billion people living with HBV worldwide, of whom 257 million persons, or 3.5% of the world’s population, have chronic HBV (1). China is one of the countries in high HBV endemicity and the prevalence of hepatitis B surface antigen (HBsAg), a specific serum marker for active HBV infection, is 7.2% (2). HBV can easily infect not only hepatocytes but also lymphocytes. The association between HBV infection and non-Hodgkin lymphomas(NHL)was first reported in 1994. Diffuse large B-cell lymphoma (DLBCL) is the most common subtype of NHL (approximately 30%–40%) (3). Several case-control studies, cohort studies and meta-analyses have since found a strong association between HBV infection and the development of DLBCL (4–9). HBV-DNA fragments and expression of HBV antigens including HBx can be detected in the lymphoid tissue of patients with HBV infection (4, 10).

At present, HBsAg-positive DLBCL is not officially recognized as a distinct subtype within the standard DLBCL classification. Despite heterogeneity exists between different clinical studies, some have suggested that HBsAg-positive DLBCL exhibits distinct clinical characteristics. HBsAg-positive patients with DLBCL have a younger median onset age and a more advanced disease at diagnosis (11, 12). Patients with DLBCL and HBV infection have poorer prognosis than those without HBV infection (13, 14). In recent years, the introduction of rituximab-containing regimens has significantly improved the survival of patients with DLBCL. Studies have demonstrated the advantages of R-CHOP over CHOP in various risk stratifications for DLBCL patients. However, many studies have not separated immunochemotherapy from chemotherapy alone when investigating the clinical significance of HBV infection, therefore, it remains unclear whether HBV infection consistently affects DLBCL patients across different treatment settings. Consequently, our study aims to compare both short-term outcomes (response rate and early progression rate) and long-term outcomes (overall survival and progression-free survival) between HBsAg-positive and HBsAg-negative patients who receive either the CHOP regimen or R-CHOP regimen, respectively, and explore the possible influencing factors of survival.

Moreover, further analysis based on genetic features is expected to have a significant impact on etiological studies, clinical treatment, and the development of new therapeutic strategies. While the genetics of DLBCL have been explored using various next-generation sequencing (NGS) methods, the unique mutational profile of HBsAg-positive DLBCL remained underexplored. Therefore, our study not only focuses on the prognostic characteristics of DLBCL patients with HBV infection but also explores the unique mutational profile of HBsAg-positive patients using NGS.

## Material and methods

### Patient selection

**Figure 1:**
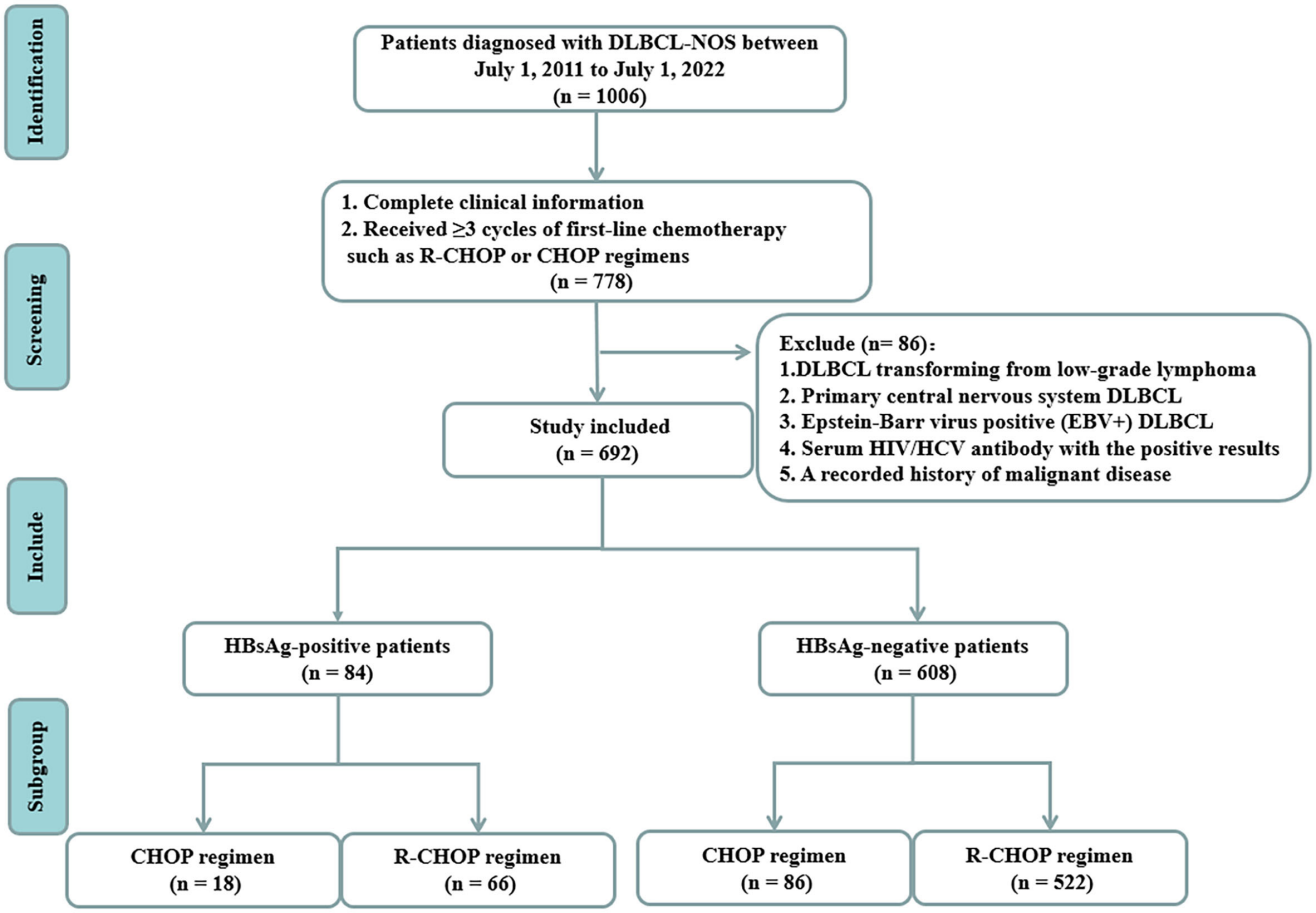
Flow chart of the study.

We retrospectively analyzed the data of all patients with DLBCL admitted to the First Hospital of Jilin University from July 2011 to July 2022. The pathological tissues were diagnosed by at least two pathologists in the pathology department of our hospital. Out of the 1006 patients initially identified, 778 patients had complete clinical information and received ≥3 cycles of first-line chemotherapy, such as CHOP or R-CHOP regimens. We excluded patients with secondary DLBCL transformed from low-grade B-cell lymphoma, primary central nervous system DLBCL, EBV infection, HCV infection, HIV infection, and a history of malignancy. Ultimately, 692 patients with DLBCL were recruited (**Figure 1**). This study was approved by the Institutional Ethical Standards of Committee at the First Hospital of Jilin University.

### Data collection

The following information was retrospectively collected from the medical charts of each patient: patient demographics, clinical backgrounds, treatment pattern and responses. All patients were tested for HBV at the time of initial diagnosis. Enzyme-linked immunosorbent assay (ELISA) was used to determine the HBV infection status, including HBsAg, hepatitis B surface antibody (anti-HBs), hepatitis B e antigen (HBeAg), hepatitis B e antibody (anti-HBe) and anti-HBc.

Abnormal liver function was defined as having alanine aminotransferase or aspartate aminotransferase levels that were more than 1.25 times the upper limit of normal. Bulky mass was characterized by one or more lesions with a size of ≥7.5 cm in their greatest dimension. The BCL2 protein was considered positive if ≥50% of the tumor cells were positive, and MYC was considered positive if ≥40% of the tumor cell nuclei were positive. HBV reactivation was defined as an increase in HBV DNA levels of more than 2 log10 from baseline levels. Among HBsAg-negative and anti-HBc positive patients, positive conversion of HBsAg was also defined as HBV reactivation.

### Treatment

The initial treatment strategy (including chemotherapy drug intensity and course) was similar between DLBCL patients with HBsAg-positive and negative in this study. In the HBsAg-positive group (n=84), 18 patients were treated with CHOP regimen and 66 patients with R-CHOP regimen. Among the 608 HBsAg-negative patients, 86 patients were treated with CHOP regimen and 522 patients with R-CHOP regimen. Serum HBV-DNA testing was performed prior to the initiation of chemotherapy and immunotherapy for all DLBCL patients, which excluded patients who had tested positive solely for anti-HBs or those who were negative for all five Hepatitis B tests. Prophylactic antiviral therapy is initiated when the treatment regimen includes medications associated with a high risk of HBV reactivation, such as glucocorticosteroids, rituximab, and anthracyclines. Antiviral prophylaxis (using lamivudine and entecavir) is maintained from initial chemotherapy to at least six months after completion of last chemotherapy for preventing HBV reactivation.

### Short- and long-term outcomes

The patients’ follow up were carried out by telephone, outpatient department visits, or electronic medical records. Overall remission rate (ORR), defined as the sum of complete remission rate (CR) and partial remission (PR) rate. POD24 was broadly defined as progression of disease within 24 months of first-line therapy (15). Overall survival (OS) was defined as time from diagnosis to death or last follow-up (November 30, 2022). Progression-free survival (PFS) was defined as time from diagnosis to disease progression, death, or last follow-up.

### Next-generation sequencing

Given the substantial time frame encompassing the original inclusion population, we aimed to explore the somatic mutation spectrum distinguishing DLBCL patients with HBsAg-positive and negative statuses. Accordingly, tumor biopsy specimens of 180 primary patients with DLBCL (excluding other viral infections) diagnosed from 2020-2022 were obtained from the Sun Yat-Sen University Cancer Center and the First Hospital of Jilin University. DNA was extracted from the formalin-fixed paraffin-embedded (FFPE) samples using the Maxwell RSC DNA FFPE Kit (Promega, USA). The samples were successfully conducted on the Novaseq (Illumina, USA) sequencing platform according to the DLBCL-related gene panel (**Supplementary Table S1**).

### Statistical analysis

General information, short-outcomes, and mutation profiles were compared using χ 2 test or Fisher’s exact test. The Kaplan–Meier method was used to plot survival curves, and the log-rank test was used for between-group comparisons. The proportional hazard assumption test was performed using the scaled Schoenfeld residuals based on a multivariable Cox proportional hazard model. Univariable and multivariable cox regression analyses were performed to identify possible influencing factors associated with OS and PFS in DLBCL patients. P ≤ 0.05 was regarded as statistically significant. Statistical tests were performed with GraphPad Prism 8.0.2 (GraphPad, La Jolla, CA, USA).

## Results

### Patient characteristics

The patients’ baseline clinical characteristics are shown in **Table 1**. 301 (43.5%) were older than 60 years; 82 (11.8%) had >2 extranodal organs; and 101 (14.6%), 203 (29.3%), 383 (55.3%), and 391 (56.5%) patients had bulky mass (≥ 7.5 cm), B symptoms, Ann Arbor stage III/IV, and IPI score ≥ 2, respectively. In this cohort, 12.1% (84/692) patients with DLBCL were HBsAg positive. The HBsAg-positive group had more frequent abnormal liver function (P = 0.003), hypoalbuminemia (P < 0.001), incidence of >2 extranodal organs (P = 0.011), and spleen involvement (P < 0.001) than the HBsAg-negative group. No significant between-group differences were observed in sex, age, serum β2-MG level, serum LDH level, bulky mass, B symptoms, Ann Arbor stage, and IPI score.

**Table 1:** Clinical characteristics of DLBCL patients with HBsAg-positive and HBsAg-negative.

Variables	All patients (n= 692)	HBsAg-positive	HBsAg-negative	P value
		patients (n= 84)	patients (n=608)	
Gender				
Male	366 (52.9%)	46 (54.8%)	320 (52.6%)	0.714
Female	326 (47.1%)	38 (45.2%)	288 (47.4%)	
Age, y				
<60	391(56.5%)	47 (56.0%)	344 (56.6%)	0.914
≥60	301(43.5%)	37 (44.0%)	264 (43.4%)	
β2-MG				
≤ULN	447 (70.0%)	49 (65.3%)	398 (70.6%)	0.353
>ULN	192 (30.0%)	26 (34.7%)	166 (29.4%)	
LDH				
≤ULN	400 (58.7%)	49 (58.3%)	351 (58.7%)	0.95
>ULN	282 (41.3%)	35 (41.7%)	247 (41.3%)	
Liver function				
Normal	582 (84.6%)	62 (73.8%)	520 (86.1%)	0.003†
Abnormal	106 (15.4%)	22 (26.2%)	84 (13.9%)	
Hypoalbuminemia				
No	419 (61.0%)	28 (33.3%)	391 (64.8%)	<0.001†
Yes	268 (39.0%)	56 (66.7%)	212 (35.2%)	
Extranodal sites				
≤2	610 (88.2%)	67 (79.8%)	543 (89.3%)	0.011†
>2	82 (11.8%)	17 (20.2%)	65 (10.7%)	
Special sites involvement				
Liver	37 (5.3%)	7 (8.3%)	30 (4.9%)	0.194
Spleen	83 (12.0%)	22 (26.2%)	61 (10.0%)	<0.001†
Bone marrow	52 (7.5%)	6 (7.1%)	46 (7.6%)	0.89
Bulky mass (≥7.5cm)	101 (14.6%)	14 (16.7%)	87 (14.3%)	0.566
ECOG				
0-1	515 (74.4%)	64 (76.2%)	451 (74.2%)	0.692
04-Feb	177 (25.6%)	20 (23.8%)	157 (25.8%)	
B symptoms				
Yes	203 (29.3%)	26 (31.0%)	177 (29.1%)	0.728
No	489 (70.7%)	58 (69.0%)	431 (70.9%)	
Ann Arbor stage				
I/II	309 (44.7%)	32 (38.1%)	277 (45.6%)	0.197
III/IV	383 (55.3%)	52 (61.9%)	331 (54.4%)	
IPI score				
0-1	301 (43.5%)	33 (39.3%)	268 (44.1%)	0.406
05-Feb	391 (56.5%)	51 (60.7%)	340 (55.9%)	

β2-MG, beta-2 microglobulin; LDH, lactate dehydrogenase; ECOG, Eastern Cooperative Oncology Group; IPI, international prognosis index. ULN, upper level of normal.

†Significant P value (<0.05).

Among the 692 cases, 450 (65.0%) cases demonstrated a non-GCB immunophenotype, whereas 173 (25.0%) cases demonstrated a GCB immunophenotype. The Ki-67 proliferation index was generally high, with ≥80% in 332 (54.4%) cases. 138/375 (36.8%) cases were tested positive for both BCL2 and MYC. Furthermore, the differences in pathological index such as cell of origin, Ki-67, BCL2, and MYC among the two groups were not significant (**Table 2**).

**Table 2:** Pathological characteristics of DLBCL patients with HBsAg-positive and HBsAg-negative.

Variables	All patients (n= 692)	HBsAg-positive	HBsAg-negative	P value
		patients (n= 84)	patients (n=608)	
Cell of origin				
GCB	173 (25.0%)	25 (29.8%)	148(24.3%)	0.56
Non-GCB	450 (65.0%)	51 (60.7%)	399 (65.6%)	
Unclassified	69 (10.0%)	8 (9.5%)	61 (10.0%)	
Ki-67				
<80%	278 (45.6%)	32 (43.8%)	246 (45.8%)	0.751
≥80%	332 (54.4%)	41 (56.2%)	291 (54.2%)	
BCL2+	507/581 (87.3%)	61/69 (88.4%)	446/512 (87.1%)	0.762
MYC+	173/402 (43.0%)	18/51 (35.3%)	155/351 (44.2%)	0.232
Both BCL2 and MYC+	138/375 (36.8%)	16/44 (36.4%)	122/331 (36.9%)	0.949

### Short-term and long-term outcomes in HBsAg-positive and negative DLBCL patients

**Figure 2:**
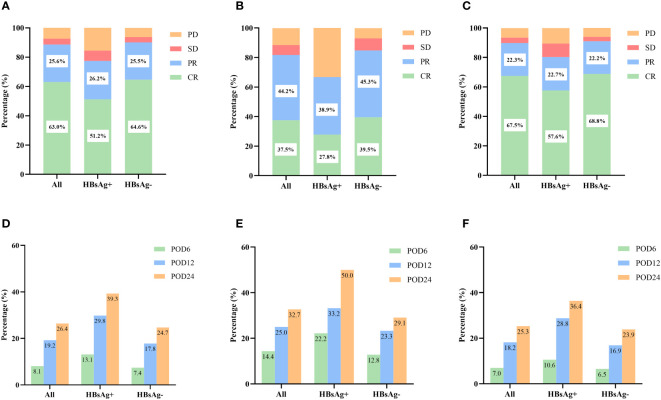
Short-term outcomes in HBsAg-positive and HBsAg-negative DLBCL patients. **(A)** Treatment response of HBsAg-positive and HBsAg-negative DLBCL patients. **(B)** Treatment response of HBsAg-positive and HBsAg-negative DLBCL patients treated with CHOP regimen. **(C)** Treatment response of HBsAg-positive and HBsAg-negative DLBCL patients treated with R-CHOP regimen. **(D)** Early progression of HBsAg-positive and HBsAg-negative DLBCL patients. **(E)** Early progression of HBsAg-positive and HBsAg-negative DLBCL patients treated with CHOP regimen. **(F)** Early progression of HBsAg-positive and HBsAg-negative DLBCL patients treated with R-CHOP regimen.

The treatment responses to the primary therapies are presented in **Figures 2A–C**. Overall, the ORRs for patients with and without HBsAg were 77.4% and 90.1%, respectively (P = 0.001), and the CR rates were 51.2% and 64.6%, respectively (P = 0.017). When receiving the same kind of therapies, HBsAg-positive patients had lower CR and ORR rates (all the p values <0.05), in either the CHOP group or R-CHOP group.

By the follow-up date, 183 patients (26.4%) had experienced disease progression, relapse, or death within 24 months after diagnosis. The incidence of POD12 (29.8% vs. 17.8%, P = 0.009) and POD24 (39.3% vs. 24.7%, P = 0.004) was higher in the HBsAg-positive group than in the HBsAg-negative group (**Figure 2D**). We also analyzed the impact of HBsAg status in patients treated with CHOP or R-CHOP on the progression of DLBCL within different periods (**Figures 2E, F**). Among patients receiving R-CHOP, the incidences of POD12 and POD24 were higher in the HBsAg-positive group than in the HBsAg-negative group (P=0.018, P=0.029). There was no significant difference in the incidence of disease progression between HBsAg-positive and HBsAg-negative patients in the CHOP group (all P values >0.05).

**Figure 3:**
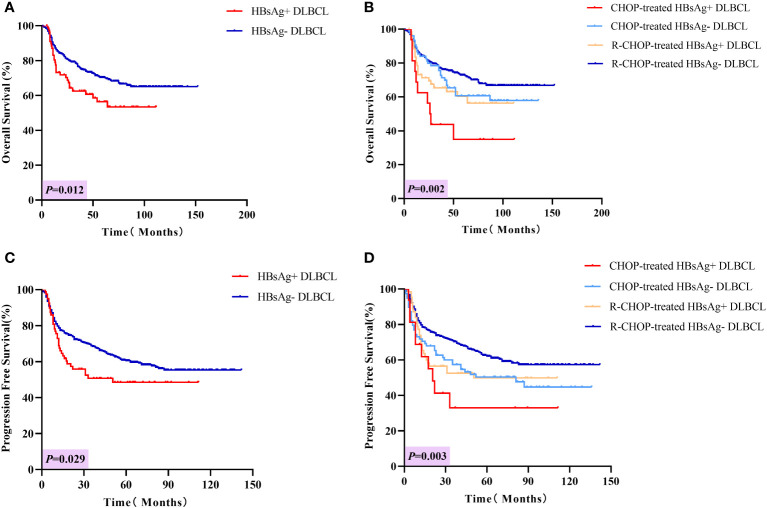
Long-term outcomes in HBsAg-positive and negative DLBCL patients. **(A)** Overall survival of HBsAg-positive and HBsAg-negative DLBCL patients. **(B)** Overall survival of HBsAg-positive and HBsAg-negative DLBCL patients treated with CHOP or R-CHOP regimen. **(C)** Progression-free survival of HBsAg-positive and HBsAg-negative DLBCL patients. **(D)** Progression-free survival of HBsAg-positive and HBsAg-negative DLBCL patients treated with CHOP or R-CHOP regimen.

The median follow-up period was 56 months. The 3-year OS rates for patients in the HBsAg-positive and HBsAg-negative groups were 62.6% and 76.4%, respectively, and the 5-year OS rates were 56.4% and 70.4%, respectively (P = 0.012, **Figure 3A**). Similarly, the PFS rates in HBsAg-positive patients were worse than those in HBsAg-negative patients (P = 0.029, **Figure 3C**). The 3-year and 5-year PFS rates in HBsAg-positive patients were 50.7% and 48.5%, respectively, and in HBsAg-negative patients were 68.5% and 60.5%, respectively. In the CHOP group, HBsAg-positive patients had a shorter OS than in HBsAg-negative patients (**Figure 3B**, P = 0.018), but the difference in PFS was not statistically significant (**Figure 3D**, P = 0.233). When receiving the R-CHOP regimen, HBsAg-negative patients were superior to HBsAg-positive patients in both OS (P = 0.038) and PFS (P = 0.040).

### Univariate and multivariate analysis of OS and PFS in all patients

Covariates such as HBsAg status, gender, β2-MG, IPI score, bulky mass, Ki-67 and rituximab-containing regimens were analyzed by Cox regression analysis (**Table 3**). In the multivariate analysis, poorer long-term outcomes, both OS and PFS, were associated with HBsAg positivity (OS: HR [95% CI] = 1.608 [1.076-2.403], P = 0.004; PFS: HR [95% CI] = 1.520 [1.054-2.162], P = 0.025), elevated β2-MG (OS: HR [95% CI] = 1.826 [1.334-2.500], P = 0.001; PFS: HR [95% CI] = 1.598 [1.198-2.078], P = 0.001), IPI score ≥ 2 (OS: HR [95% CI] = 3.389 [2.292-5.010], P < 0.001; PFS: HR [95% CI] = 2.749 [1.999-3.780], P < 0.001). Rituximab-containing was associated with better OS and PFS (OS: HR [95% CI] = 0.591[0.411-0.850], P = 0.005; PFS: HR [95% CI] = 0.573[0.412-0.796], P = 0.001). Notably, in the subgroup analysis, HBsAg positivity (OS: HR [95% CI] = 2.511 [1.214-5.192]) was only associated with poorer OS in the CHOP group. Whereas in the R-CHOP group, HBsAg positivity was associated with both poorer OS and PFS (OS: HR [95% CI] = 1.672 [1.050-2.665], P = 0.030; PFS: HR [95% CI] = 1.536 [1.013-2.331], P = 0.043).

**Table 3:** Univariate and multivariate analysis of OS and PFS in all patients.

Characteristics	Variable	Reference	OS				PFS			
			Univariate		Multivariate		Univariate		Multivariate	
			P value	HR (95% CI)	P value	HR (95% CI)	P value	HR (95% CI)	P value	HR (95% CI)
All patients										
HBsAg status	positive	negative	0.004†	1.625 (1.105-2.390)	0.020†	1.608 (1.076-2.403)	0.004†	1.625 (1.163-2.271)	0.025†	1.520 (1.054-2.162)
Gender	male	female	0.183	1.218 (0.911-1.628)			0.126	1.215 (0.947-1.558)		
β2-MG	>ULN	≤ULN	<0.001†	2.795 (2.076-3.762)	0.001†	1.826 (1.334-2.500)	<0.001†	2.242 (1.729-2.908)	0.001†	1.598 (1.198-2.078)
Bulky mass	≥7.5cm	<7.5cm	0.025†	1.504 (1.053-2.148)	0.462	1.148 (0.794-1.660)	0.025†	1.434 (1.047-1.964)	0.378	1.158 (0.835-1.606)
IPI score	2-5	0-1	<0.001†	4.238 (2.954-6.081)	<0.001†	3.389 (2.292-5.010)	<0.001†	3.064 (2.304-4.075)	<0.001†	2.749 (1.999-3.780)
Ki-67	≥80%	<80%	0.445	1.129 (0.828-1.539)			0.961	0.993 (0.761-1.297)		
Rituximab-containing	yes	no	0.026†	0.672 (0.473-0.954)	0.005†	0.591 (0.411-0.850)	0.009†	0.661 (0.484-0.901)	0.001†	0.573 (0.412-0.796)
CHOP group										
HBsAg status	positive	negative	0.030†	2.223 (1.082-4.570)	0.013†	2.511 (1.214-5.192)	0.242	1.518 (0.755-3.052)		
Gender	male	female	0.939	1.024 (0.549-1.910)			0.166	1.493 (0.847-2.630)		
β2-MG	>ULN	≤ULN	0.187	1.567 (0.804-3.054)			0.024	2.011 (1.094-3.697)	0.451	1.283 (0.671-2.452)
Bulky mass	≥7.5cm	<7.5cm	0.787	1.113 (0.513-2.416)			0.949	0.977 (0.474-2.012)		
IPI score	2-5	0-1	0.001†	3.133 (1.603-6.123)	<0.001†	3.324 (1.695-6.522)	<0.001†	3.048 (1.676-5.543)	0.001†	3.278 (1.663-6.461)
Ki-67	≥80%	<80%	0.654	1.163 (0.601-2.249)			0.696	1.124 (0.624-2.025)		
R-CHOP group										
HBsAg status	positive	negative	0.042†	1.588 (1.018-2.478)	0.030†	1.672 (1.050-2.665)	0.015†	1.604 (1.094-2.352)	0.043†	1.536 (1.013-2.331)
Gender	male	female	0.192	1.243 (0.896-1.723)			0.295	1.160 (0.879-1.530)		
β2-MG	>ULN	≤ULN	<0.001†	3.176 (2.272-4.439)	0.001†	2.180 (1.531-3.104)	<0.001†	2.317 (1.737-3.091)	<0.001†	1.673 (1.230-2.275)
Bulky mass	≥7.5cm	<7.5cm	0.025†	1.584 (1.061-2.365)	0.869	1.036 (0.680-1.572)	0.015†	1.541 (1.086-2.185)	0.468	1.146 (0.793-1.656)
IPI score	2-5	0-1	<0.001†	4.766 (3.097-7.335)	<0.001†	3.539 (2.230-5.614)	<0.001†	3.213 (2.316-4.458)	<0.001†	2.615 (1.828-3.740)
Ki-67	≥80%	<80%	0.504	1.128 (0.792-1.606)			0.680	0.939 (0.695-1.268)		

β2-MG: beta-2 microglobulin, IPI: international prognosis index.

†Significant P value (<0.05).

### HBV-related events

Among the HBsAg-positive patients, 38 (45.2%) patients presented detectable baseline HBV DNA, 12 patients (14.3%) developed HBV reactivation. In the HBsAg-negative and anti-HBc positive patients (n=159), 6 patients (3.8%) developed HBV reactivation. All HBV reactivation occurred during immunochemotherapy rather than after the cessation of immunochemotherapy. No HBV-related fatal liver failure and liver cancer occurred.

### Somatic mutation spectrum of patients with DLBCL under different HBsAg status

**Figure 4:**
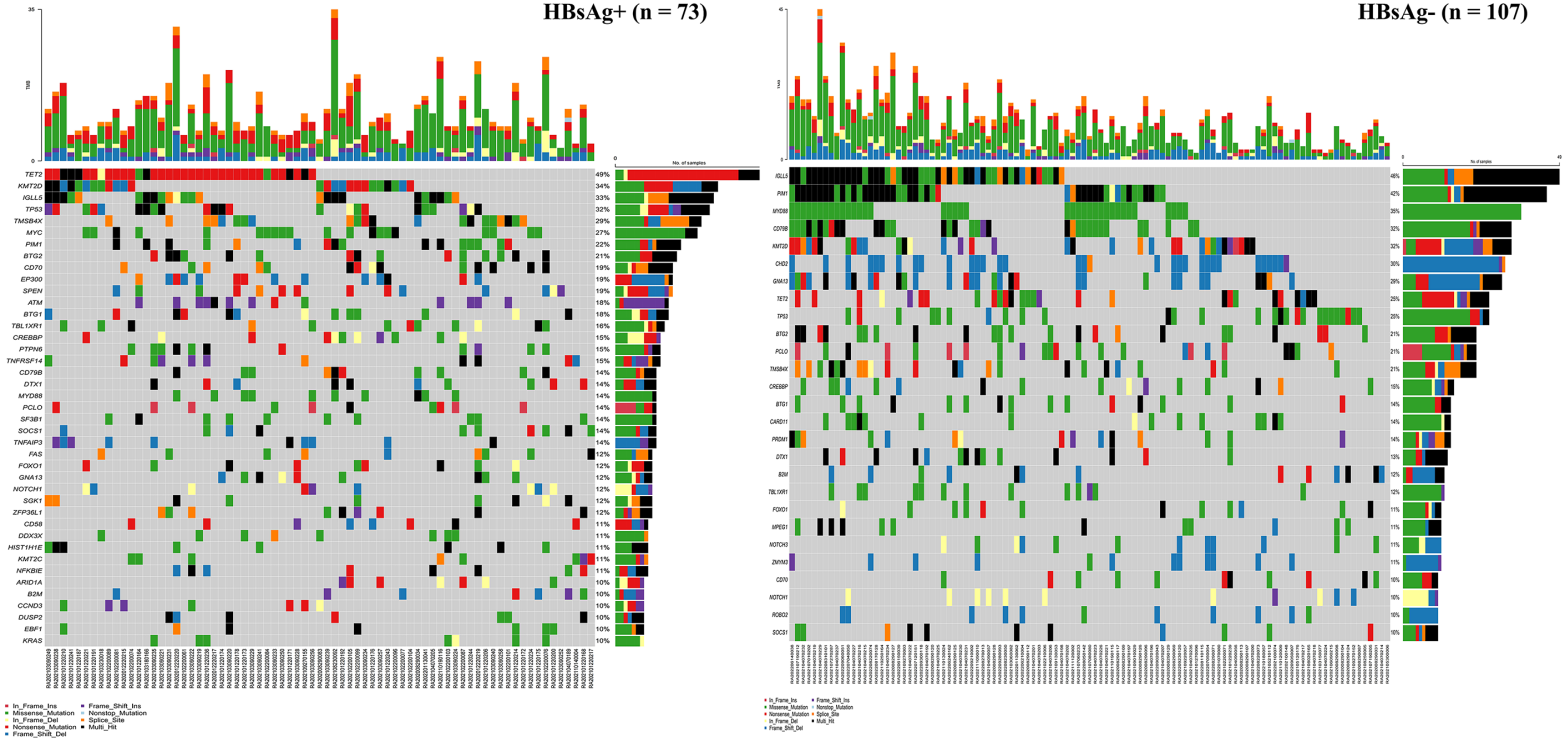
Somatic mutation spectrum in HBsAg-positive and HBsAg-negative DLBCL patients.

There were 73 HBsAg-positive and 107 HBsAg-negative cases with DLBCL. All patients had at least one mutated gene. As presented in **Figure 4**, in the HBsAg-positive group, the most frequently mutated (≥15%) genes were TET2, KMT2D, IGLL5, TP53, TMSB4X, MYC, PIM1, BTG2, CD70, EP300, SPEN, ATM, BTG1, TBL1XR1, CREBBP, PTPN6, and TNFRSF14. Thirteen genes were confirmed to be frequently mutated (≥15%) in the HBsAg-negative group, including IGLL5, PIM1, MYD88, CD79B, KMT2D, CHD2, GNA13, TET2, TP53, BTG2, PCLO, TMSB4X, and CREBBP.

**Figure 5:**
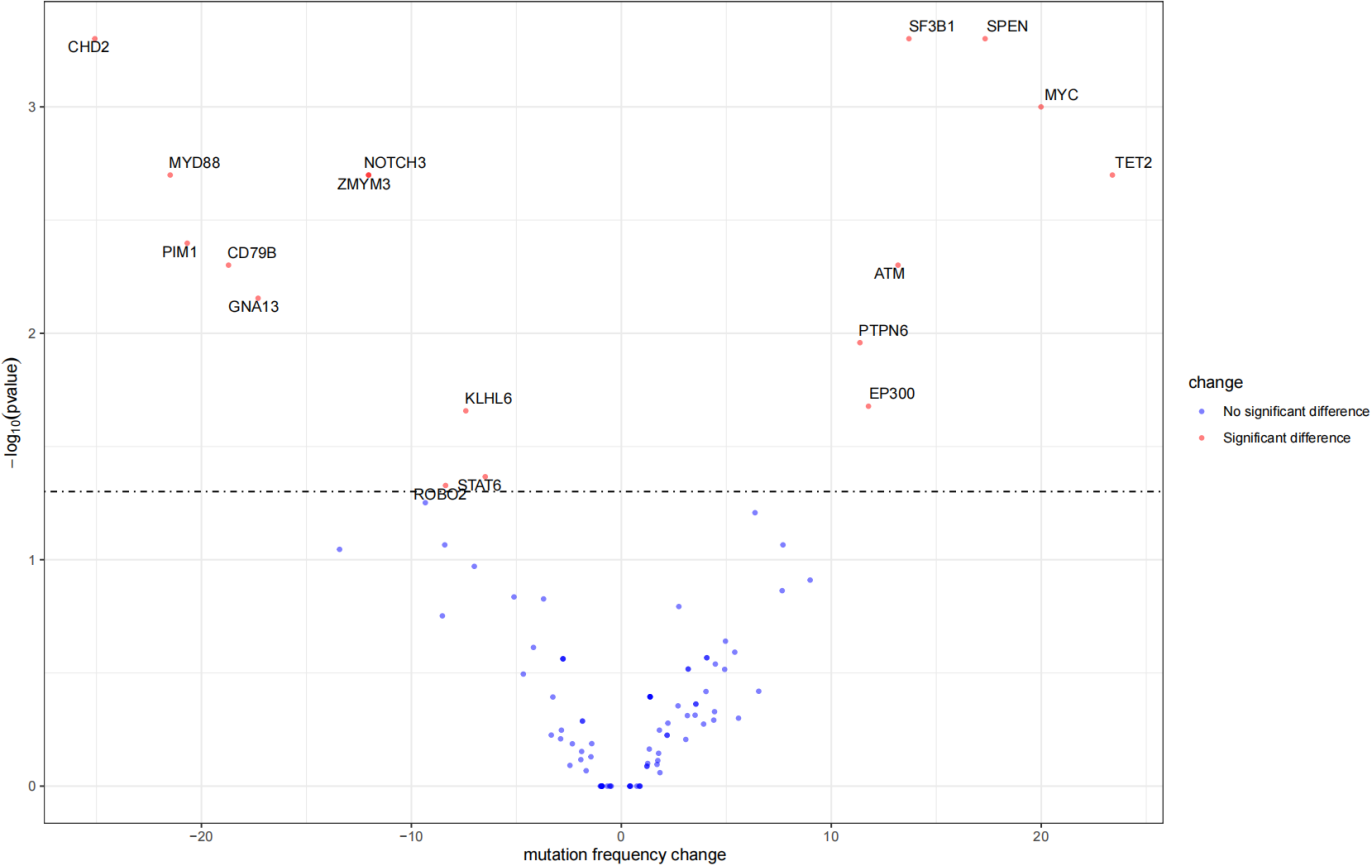
Differential mutation genes in HBsAg-positive DLBCL patients compared to HBsAg-negative patients.

**Figure 6:**
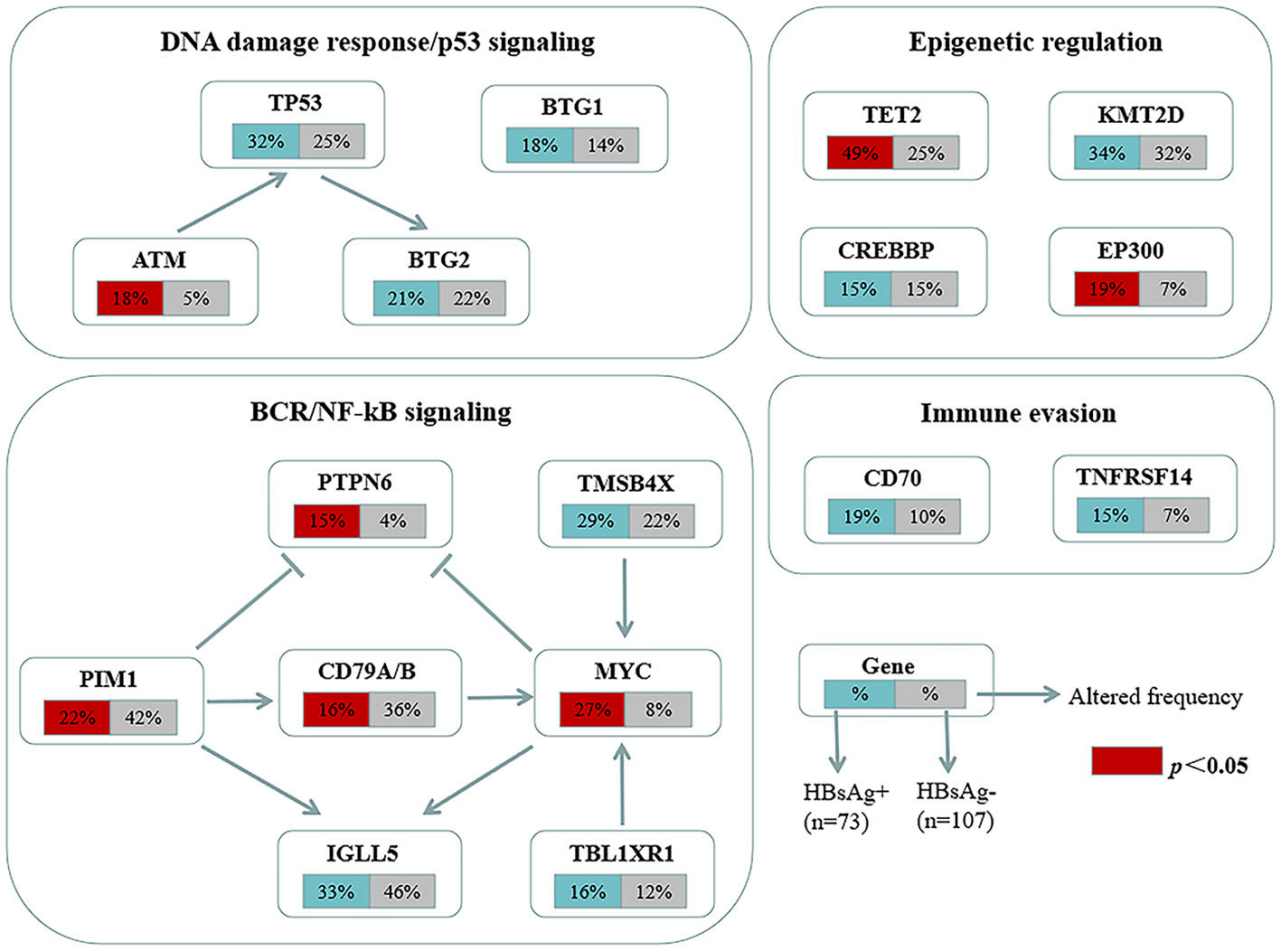
Key signaling pathways that are affected by frequent somatic mutations in HBsAg-positive DLBCL patients. The frequency of mutated genes in HBsAg-positive and HBsAg-negative DLBCL are shown under the gene names.

The mutation profiles in HBsAg-positive and HBsAg-negative DLBCL were subsequently compared. First, seven genes had a higher mutation frequency in the HBsAg-positive group than in the HBsAg-negative group, including TET2, MYC, SPEN, SF3B1, ATM, EP300, and PTPN6. Second, some mutation targets in DLBCL, such as CHD2, MYD88, PIM1, CD79B, and GNA13 were less frequently mutated in the HBsAg-positive group (**Figure 5**). There is a high-frequency set of mutated genes in HBsAg-positive DLBCLs that may affect multiple key pathways involved in lymphoma development, such as epigenetic regulation, DNA damage repair, BCR/NF-kB, and immune evasion (**Figure 6**).

## Discussion

Several epidemiologic and clinical studies have proposed that DLBCL patients with concurrent HBV infection might constitute a unique subgroup, distinguished by distinct clinical characteristics. Based on a review of existing literature, this is the largest cohort study to attempt to clarify the prognostic implications of HBV infection in patients with DLBCL across different treatment settings. The HBsAg-positive rate among patients with DLBCL in our study was 12.1%, which was similar to or lower than the rates observed in previous studies from China (11.6%–30.9%) (16–19) but significantly higher than the general population and patients with solid tumors other than primary liver cancer (20).

In our study, HBsAg-positive patients have unique clinical features and are more likely to experience abnormal liver function, hypoalbuminemia, and spleen involvement at the time of initial diagnosis, consistent with previous studies (16, 19). Our novel finding was that the incidence of extranodal involved organs >2 was significantly higher in the HBsAg-positive group than in the HBsAg-negative group. This suggests that DLBCL with HBsAg-positive typically exhibits aggressive biological behavior, including a higher migration, and invasive ability of HBV-infected lymphoma. Unlike previous studies, no significant difference was observed in the IPI scores between the two groups, indicating that the risk stratification of patients with DLBCL at initial diagnosis did not differ significantly and that baseline status may not be a major risk factor affecting the adverse prognosis of HBsAg-positive patients with DLBCL.

In the current rituximab era, although the CR rate was significantly higher in the R-CHOP group than in the CHOP group. We observed that HBsAg-positive DLBCL patients were less sensitive to the drug than the HBsAg-negative patients in either the CHOP group or R-CHOP group, which is a significant factor contributing to treatment failure, refractory relapse, and poor prognosis. HBV infection persistently activates the NF-kB pathway, leading to the upregulation of multidrug resistance gene expression in hepatocellular carcinoma cells, resulting in reduced sensitivity to a variety of chemotherapeutic agents (21, 22). However, the specific mechanism by which HBV infection confers chemoresistance in DLBCL requires further investigation. Studies have shown that overexpression of the core component of HBV (HBx) can attenuate the sensitivity of GCB-subtype DLBCL cells to second-line chemotherapeutic agents such as MTX or Ara-C. Mechanism studies have shown that HBx conferred resistance to the S-phase arrest-inducing chemotherapeutics by specifically inhibiting the phosphorylation of checkpoint kinase 2 (CHK2, a key DNA damage response protein) in DLBCL cells (23). Additionally, an experimental study suggested that HBx directly upregulated the expression of lncNBAT1, which can interact with STAT1 to prevent its enrichment at the promoter region of the functional target gene apolipoprotein B mRNA editing enzyme catalytic subunit 3A (APOBEC3A), inhibiting the expression of APOBEC3A and inducing resistance to MTX in DLBCL cells (24).

Early progression, such as POD24, serve as a robust indicator of poor survival in DLBCL. Notably, patients with DLBCL who do not experience POD24 had an overall survival rate equivalent to that of the age- and sex-matched general population (25). However, limited research has explored the association of HBV infection with early progression in DLBCL. Our data indicated that the incidences of both POD12 and POD24 were higher in the HBsAg-positive group compared to the HBsAg-negative group. This highlights that early progression is an important feature of HBsAg-positive patients with DLBCL, which may be attributed to the lymphoma’s biological aggressiveness and potential resistance to first-line chemotherapeutic agents. In the R-CHOP group, HBsAg positivity was associated with both inferior OS and PFS. Even in the context of the current rituximab era, DLBCL patients with HBV infection have improved response to first-line chemotherapy differs significantly for compared to the CHOP era, HBsAg positivity remains an important adverse factor affecting long-term outcomes. As we know, rituximab-containing regimens could induce a profound immunosuppression and are associated with a higher incidence of HBV reactivation and fatal liver dysfunction (26, 27). However, the reactivation rate among HBsAg-positive patients in our study was generally lower than in previous studies (28, 29). Beyond HBV reactivation, HBV infection may negatively influence patient prognosis through other pathways in R-CHOP group.

The unique mutational profile of HBsAg-positive DLBCL remains underinvestigated (30, 31). We observed a distinct set of genetic alterations in the genomes of patients with HBsAg-positive DLBCL, which included frequent mutations in TET2, MYC, SPEN, SF3B1, ATM, EP300, and PTPN6. A study showed that potentially off-targets of activation-induced cytidine deaminase were preferentially mutated in the HBsAg-positive group, such as KLF2, TMSB4X, CD70, Bcl6, ZFP36L1, CXCR4, and FOXO1. TET2, EP300, and MYC have not been appreciated previously as significant mutation targets in HBsAg-positive DLBCL (30). Therapies targeting these genes may help suppress tumor growth. The genetic changes associated with HBsAg-positive DLBCL primarily affect several pathways, such as DNA damage repair/p53 signaling, BCR/NF-kB signaling, and immune evasion. Ye et al. also discovered that chronic HBV infection within the lymphoma tumor microenvironment and its interaction mechanisms can significantly impact tumor cell immune evasion in DLBCL (31). Notably, the HBsAg-positive group exhibited a decreased mutation frequency of CD79B, a key molecule of the BCR pathway; thus, HBV infection may affect the immune escape mechanism of tumor cells and, thus, the mutational selection of CD79B.

The main limitations of our study encompass its relatively small sample size for sequencing, and limited generalizability. Another limitation is the absence of data pertaining to the dynamic alterations in liver function, as per the Common Terminology Criteria for Adverse Events (CTCAE) grade, during the course of chemotherapy. Looking ahead, our future studies will prioritize investigating the connection between HBV infection and chemoresistance, delving into the underlying immunobiological mechanisms, and exploring potential epigenetic alterations.

In conclusion, our comprehensive investigation revealed the clinical significance of HBV infection in individuals diagnosed with DLBCL. The HBsAg-positive group should represent a distinct subgroup with unique clinical features (more frequent extranodal involvement, higher incidence of abnormal liver function and hypoalbuminemia), prognostic features (reduced sensitivity to first-line chemotherapeutic agents, higher rates of early progression, shorter OS and PFS), and genetic features (frequent mutations in MYC, ATM, PTPN6 and epigenetic regulatory genes) compared to the HBsAg-negative group. These findings suggest that standard therapy may be insufficient for HBsAg-positive patients with DLBCL and that novel treatment strategies should be developed based on a better understanding of the underlying mechanisms of chemoresistance.

## Data availability statement

The raw sequence data reported in this paper have been deposited in the Genome Sequence Archive (Genomics, Proteomics & Bioinformatics 2021) in National Genomics Data Center (Nucleic Acids Res 2022), China National Center for Bioinformation / Beijing Institute of Genomics, Chinese Academy of Sciences (GSA-Human: HRA005906) that are publicly accessible at https://ngdc.cncb.ac.cn/gsa-human.

## Ethics statement

The studies involving humans were approved by the Human Ethics Committee of the First Hospital of Jilin University. The studies were conducted in accordance with the local legislation and institutional requirements. Written informed consent for participation was not required from the participants or the participants’ legal guardians/next of kin in accordance with the national legislation and institutional requirements.

## Author contributions

ZZ: Data curation, Investigation, Methodology, Software, Writing – original draft. WG: Formal analysis, Project administration, Resources, Writing – review & editing. XW: Investigation, Methodology, Writing – original draft. BW: Data curation, Writing – original draft. JL: Methodology, Writing – original draft. HW: Data curation, Writing – original draft. ZL: Data curation, Writing – original draft. YH: Formal analysis, Resources, Writing – review & editing. KY: Supervision, Writing – review & editing. OB: Resources, Supervision, Writing – review & editing, Project administration.

## Supplementary material

Supplementary Table S1 DLBCL-related gene panel.
